# Anatomical structure interpretation of the effect of soil environment on fine root function

**DOI:** 10.3389/fpls.2022.993127

**Published:** 2022-08-30

**Authors:** Tianyi Li, Jingjing Ren, Wenchun He, Yu Wang, Xiaochen Wen, Xiao Wang, Mengting Ye, Gang Chen, Kuangji Zhao, Guirong Hou, Xianwei Li, Chuan Fan

**Affiliations:** ^1^College of Forestry, Sichuan Agricultural University, Chengdu, China; ^2^National Forestry and Grassland Administration Key Laboratory of Forest Resources Conservation and Ecological Safety on the Upper Reaches of the Yangtze River and Forestry Ecological Engineering in the Upper Reaches of the Yangtze River Key Laboratory of Sichuan Province, Chengdu, China

**Keywords:** fine root, anatomical structure, root function, root order, soil environment, *Cupressus funebris*

## Abstract

Fine root anatomy plays an important role in understanding the relationship between fine root function and soil environment. However, in different soil environments, the variation of fine root anatomical structure in different root sequences is not well studied. We measured the soil conditions and anatomical structure characteristics (root diameter, cortical tissue, vascular tissue and xylem) of fine roots of *Cupressus funebris* in four experimental sites, and analyzed each level of fine roots separately. We link these data to understand the relationship between fine root anatomy and soil conditions. We found that the anatomical structure of fine roots is closely related to soil environmental factors. The fine roots of lower root order are mainly affected by soil nutrients. Among them, the cortical tissue of first-order fine roots was positively correlated with potassium and phosphorus, but negatively correlated with nitrogen, while second- and third-order fine roots was positively correlated with soil total potassium and negatively correlated with nitrogen and phosphorus. For the fine roots of high root order, the cortical tissue disappeared, and the secondary vascular tissue was mainly affected by soil moisture. In addition, we also found that the division of fine root functional groups is not fixed. On the one hand, the function of third-order fine roots will slip. For example, the decrease of soil moisture will promote the transformation of third-order fine roots into transport roots, and the reduction of nitrogen will promote the transformation of third-order fine roots into absorption roots to fix nitrogen. This transformation strategy can effectively prevent the restriction of soil nutrients on plant growth. On the other hand, with the change of habitat, the first- and second-order fine roots are still the absorbing root, and the fourth- and fifth-order fine roots are still the transport root, but the efficiency of absorption and transport will be affected. In conclusion, our findings emphasize the fine roots in different soil environment to show high levels of plasticity, shows that fine root anatomical structure changes may make plants, and reveals that the fine is just order of reaction and its mechanism in the soil environment.

## Introduction

Fine roots are the most physiologically active plant components of the root system (Cuneo et al., 2020). With short life span and fast turnover rate (Hu et al., 2021), fine roots are mainly responsible for absorbing a large amount of water and nutrients and carrying out carbon (C) and nitrogen (N) cycling (Zeng et al., 2020; Endo et al., 2021; Huaraca et al., 2021), which ensuring an adequate supply of water and nutrients for plant photosynthesis, growth, and maintenance (Freschet and Roumet, 2017). Fine root anatomical structure is the direct expression of root growth and development, and there is a strong relationship between anatomical structure and physiological function (Wang et al., 2019; Zhou et al., 2022). The anatomical structure of fine roots is mainly composed of cortex and stele (Guo et al., 2008), which reflects two main functions of fine roots: absorption and transport (Kong et al., 2014; Mao et al., 2018; Wang et al., 2019). In recent years, root functional ecology and physiological ecology have attracted much attention (Freschet et al., 2021). However, despite the recognition of functional differences, few studies have involved anatomical structures, especially the anatomical structures of fine roots in different soil conditions. Due to the difficulty of sampling and the complexity of research, the research on fine root anatomical structure and its relationship with environment and related physiological activities is not sufficient.

Root order is vital for understanding the root function in fine root system (Guo et al., 2008). Traditionally, fine roots generally consist of 2–5 root orders (Freschet and Roumet, 2017). Specifically, roots farthest from the main root axis of the root system, being root tips with no more branches, were defined as the 1-order fine root; the parent root of the 1-order fine root was defined as the 2-order fine root; and so on, until the 5-order fine root was reached. In addition, roots borne on a higher order fine root without branches were also classified as a 1-order fine root (He et al., 2022). Many studies have emphasized the differences of anatomical structures of fine roots in different branch orders (Liu et al., 2019). From first- to fifth-order fine roots, the cortex gradually decreases (or disappears), while the stele gradually increases (Guo et al., 2008; Endo et al., 2021). Later, the researchers found that the functional characteristics of fine roots with different branch order can be directly expressed by the internal anatomical structure of fine roots (Guo et al., 2008). Specifically, transport fine roots are generally high order, have thicker diameter and greater ratio of stele to root diameter. In contrast, absorptive fine roots are low order, have thinner diameter and lower ratio of stele to root diameter (Wang et al., 2015). That is to say, from 1- to 5-order fine roots, the absorption capacity of fine roots gradually decreases, while the transport capacity gradually increases (McCormack et al., 2015). In fact, this change in the physiological function of advanced fine roots is related to secondary development (Wang et al., 2019), such as the development of cork periderm, endodermis (Wells and Eissenstat, 2002; Hishi, 2007). This is because with the increase of root order, fine roots require greater axial transport capacity. These changes in anatomical structure reduced the ability of roots to absorb water and nutrients, and increased the transport efficiency of higher roots (Wang et al., 2019).

Ecological factors can affect the anatomical structure of fine roots (Wang et al., 2015; Huaraca et al., 2021). Temperature can affect not only plant roots directly, but also fine roots indirectly by affecting soil nutrient conditions (Sorensen et al., 2008). For example, at low temperature, plants get more water and nutrients by increasing the diameter of fine roots and producing more absorbing roots (Zadworny et al., 2016), so as to improve the adaptability of fine root to environmental change. It is also worth noting that fine root growth also increases significantly at high temperature (Malhotra et al., 2020). Present day science indicates that the stimulation of photosynthesis at high temperature and the prolongation of growing season were beneficial to root growth (Wang et al., 2021), and the response mechanism of fine roots to climate change was further clarified. In addition, soil moisture also has a certain effect on the anatomical structure of fine roots. The absorption of water by the root first involves the radial water transport from the soil solution to the root column, and then flows upward into the shoot through the xylem catheter (Ouyang et al., 2020). The response of fine roots to drought involves the complex relationship between anatomical structure and function (Barrios-Masias et al., 2015; Reingwirtz et al., 2021). Studies have shown that plants in arid environments will carry out drought avoidance strategies (Freschet et al., 2017). Under drought conditions, the roots need to absorb enough soil water to meet the transpiration needs of the canopy (Cuneo et al., 2020). At this time, the fine root enhances the absorptive capacity by reducing the diameter (Nikolova et al., 2020). The results showed that the root system adapted to soil water stress changed the anatomical structure, so as to achieve the best growth state.

Further, the fine root anatomical structure also changes with the local soil environment (Hogan et al., 2020). Soil bulk density is also likely to be an important driver of fine root trait variation (Eissenstat et al., 2015), it usually has a positive effect on the fine root diameter (Clark et al., 2003). Plants with higher fine root diameter have greater root elongation performance in denser soils (Freschet et al., 2017), whereas the contrary may be true in less dense soils (Eissenstat et al., 2015). This is most likely because a smaller soil bulk density provides a lower mechanical impedance, which promotes root dispersal (Cao et al., 2021).

At the same time, it is found that larger soil pores and fine root density can create more preferential flow paths for stem flow and natural precipitation (Johnson and Lehmann, 2006), promoting plants to absorb more water and nutrients. Additionally, about soil nutrients (nitrogen, phosphorus, potassium) and anatomical structure of fine roots, lack of nutrients in the soil usually leads to root elongation and structural development (Hogan et al., 2020). Nitrogen (N) is arguably the most studied element in plant function and growth (Hogan et al., 2021). In the soil with high concentration of N, the metabolic activity of fine roots is stronger (Bardgett et al., 2014), such as root elongation (Eissenstat et al., 2015), nutrient uptake (Freschet and Roumet, 2017), proton release and active C exudation (Jones et al., 2009). With the increase of N content, the cortical thickness of the first three grades of fine roots decreased significantly (Chen et al., 2010), indicating that the increase of N content could promote the transport function of fine roots. In general, N deficient soil leads to increased lateral root elongation, while phosphorus (P) deficient soil leads to more branching roots (Giehl et al., 2013). On the one hand, the decrease of soil available phosphorus will reduce the diameter of fine roots, on the other hand, the surface area of fine roots will increase (Ushio et al., 2015). Therefore, fine roots can still absorb water and nutrients to the maximum extent. Several other micronutrients, such as calcium (Ca), magnesium (Mg), sulfur (S), and other trace metals, are required in small amounts by plants but rarely limit their physiological functions. For example, calcium, if they are in compounds that are phloem-insoluble or too large for phloem transport, since the amount of calcium in the soil has relatively little effect on plants (Hogan et al., 2021). In general, the root diameter decreased in areas with low soil nutrients (Hogan et al., 2020). These studies show that with the changes of soil environment, the distribution and morphological structure of the fine root system will change obviously (Hertel and Schöling, 2011; Moser et al., 2011; Girardin et al., 2013; Mao et al., 2015).

Taken together, these studies support the notion that fine roots can adjust a variety of structural and functional traits to adapt to different soil conditions, which is closely related to the changes of fine root anatomical structure and is also vital for the growth of trees and the sustainable development of forests. Considering that the variation of fine root anatomical structure will lead to the change of fine root function, we suppose the fine root function group will change in different soil environment. Meanwhile, although some scholars have studied the anatomical structure based on artificially changing some soil physical and chemical indexes, how do fine roots adapt to the long-term natural growth of soil by changing the anatomical structure (Nikolova et al., 2020)? It’s not clear yet. In this case, it is crucial to determine how fine root anatomical structures of different root orders adapts to and responds to changes in soil environment and the relationship between fine root anatomical structure and function.

Our study is based on *Cupressus funebris* of several distribution areas including Suining, Mianyang, Deyang and Guangan, which represented the hilly areas in the middle of Sichuan. We excavated roots by the soil monolith method (Wang et al., 2015). In this study, we attempted to find out the changing trend and response mechanism of anatomical structure under different soil environmental factors by measuring the anatomical structure of first- to fifth-order fine roots between different sites and the soil physical and chemical properties of the corresponding sites. Based on this, we propose two hypotheses: (1) the fine root anatomical structure of *C. funebris* in four sites showed plasticity, the response of fine root anatomical structure of different root orders to soil environmental factors is not consistent, (2) due to changes in anatomical structure, the division of fine root functional groups is not fixed.

## Materials and methods

### Study site’ description

**Figure 1:**
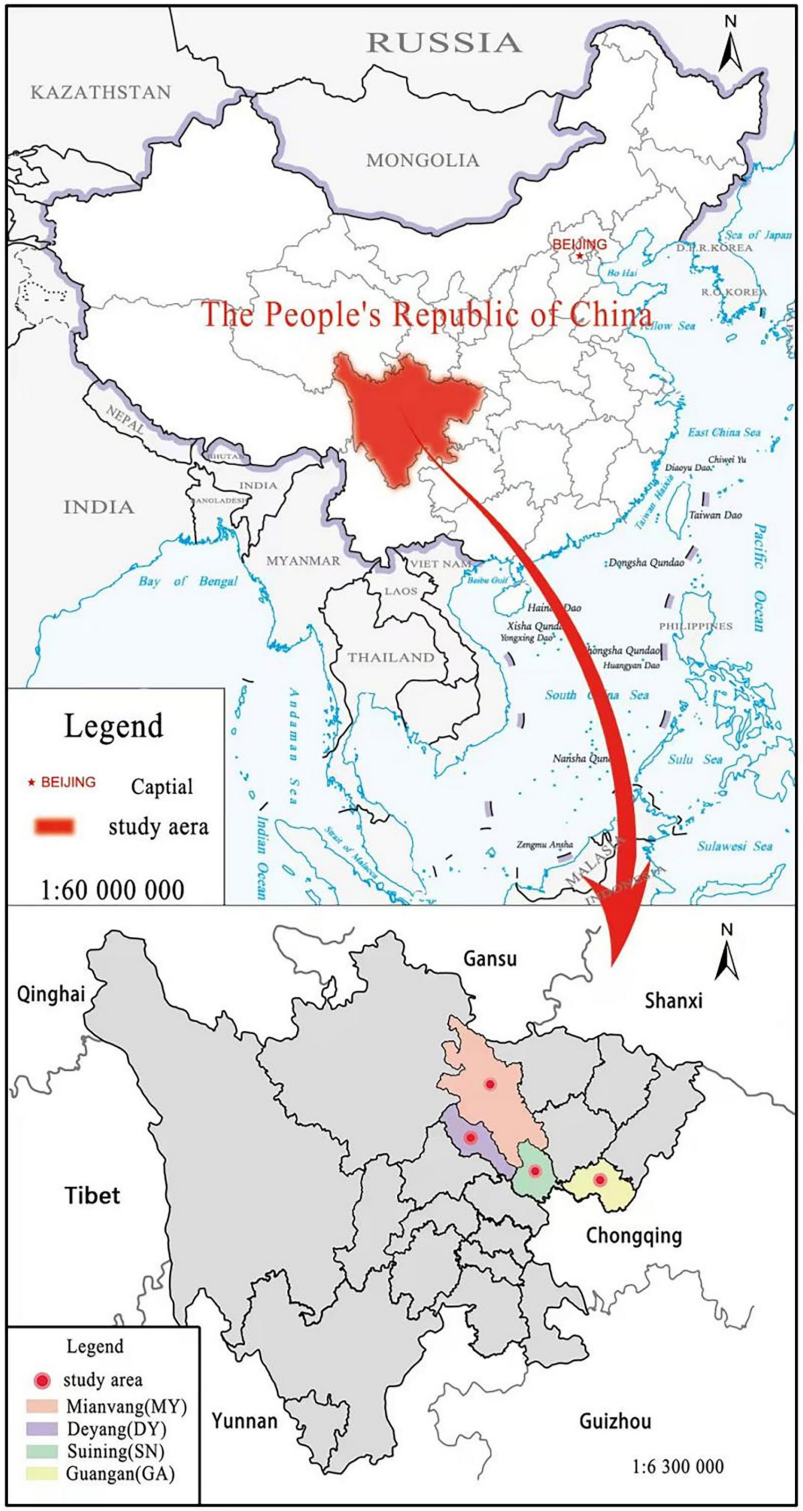
Distribution map of the study sites. Location map of Sichuan province and the four study sites in Sichuan province: Mianyang (MY), Deyang (DY), Suining (SN), Guangan (GA).

We selected four sampling sites with large differences in soil environment in rural mountainous areas, namely, Jingyang District of Deyang city (DY), Anju District of Suining City (SN), Yanting County of Mianyang City (MY) and Huaying City of Guang’an City (GA) to represent shallow hill area, middle hill area, deep (high) hill area and low mountain area successively (Figure 1). These four sites are all ecological groups of single-species *C. funebris* plantations, located in the subtropical humid monsoon climate zone. The forest topsoil thickness is about 30 cm, and the age of *C. funebris* is 25–30 years old. We choose this age of *C. funebris* because at this time the growth of *C. funebris* is relatively stable, which is more conducive to the determination of anatomical structure indicators. The dominant species of understory plants were *Myrsine africana*, *Vitex negundo* and *Coriaria nepalensis*. The detailed nature of the test site is shown in Table 1.

**Table 1:** Basic geographic information of four test sites.

Item	Site			
	Suining	Deyang	Mianyang	Guangan
Latitude	104°25′41′	105°26′39′	105°31′57′	106°42′13′
Longitude	31°04′01′	31°15′54′	30°24′37′	30°03′53′
Mean annual precipitation/(mm)	930	893	880	1,200
Mean annual temperature/(°C)	17.4	16.5	17.3	16.0
Sunshine duration/(h)	1116.5	1057.6	1205.2	1,340
Annual frost-free period (d)	296	274	294	319
Altitudes/(m)	530	415	378	731
Slope gradient/(°)	23	27	25	28
Slope aspect	Southwest	Southwest	Southeast	Southeast
Soil type	Purple soil	Purple soil	Purple soil	Yellow soil
Soil pH	8.31	7.92	8.16	7.42
Stand age/(a)	25 ~ 30	25 ~ 30	25 ~ 30	25 ~ 30
Stand density (plant·hm^−2^)	1,515	1,470	1,695	1740
Crown density	0.8	0.7	0.8	0.7
Average tree height/(m)	7.5	8.0	9.0	7.5
Average DBH/(cm)	11.5	12.0	12.5	10.5
Understory plants	*Smilax china*, *Coriaria nepalensis*	*Myrsine africana*, *Commelina communis*	*Smilax china*, *Myrsine africana*, *Coriaria nepalensis*	*Myrsine africana*, *Coriaria nepalensis*, *Ficus tikoua*

### Experimental design, root sampling and anatomical structure analysis

Our experimental study encompasses four sites. According to the preliminary investigation (stand density, tree height, diameter at breast height (DBH), natural conditions, etc.), in each site, we randomly selected three standard plots with light disturbance, well-developed natural vegetation, canopy density of about 0.8 and similar vertical space as standard plots (He et al., 2022).Then, 3 standard plots × 4 sites, for a total of 12 established in this study, with a standard plot area of 600 m^2^ (20 m × 30 m).

The fine roots sampling took place in July 2017. According to DBH and tree height, we selected 3 relatively scattered trees in each plot as standard trees. Then, we selected four directions in each standard wood (there was no obvious direction in the fine root of *C. funebris* wood after the previous study), and determined that it was the main root of the plant according to the direction of the root, dug carefully along the main root, and collected a complete root segment containing 1- to 5-order fine roots born on the main root. In each sample sites, we selected 18 1-order fine roots, 18 2-order fine roots, 15 3-order fine roots, 6 4-order fine roots, and 6 5-order fine roots, and four sites have a total of 756 roots. After collection, the fine roots samples were stored in a refrigerator at low temperature (0–4°C) and transported to the laboratory as soon as possible.

In the laboratory, we carefully separate the fine roots from the soil and gently removed soil particles and debris that cling to the roots. The root samples were washed with deionized water at room temperature and the fine roots were stored as anatomical samples in formalin-aceto-alcohol (FAA) fixation solution. If the root segment was too long, we processed it to about 1 cm to facilitate subsequent experimental operations. Then we use the paraffin technique to make fine root cross sections, and all root cross sections were stained with safranin-fast green. After gradient alcohol dehydration (alcohol concentrations of 70, 85, 95 and 100%), safranin-fast green staining, wax-impregnation (using xylene as a drying solvent at 45°C in an oven for 20 h), and paraffin embedding, fine roots were cut into thin sections, and dewaxed to achieve transparency. Finally, 8 μm thick sections were prepared for analysis (Liu et al., 2019). For each root cross section, eight sampling points on each slice with complete root morphology were selected and photographed under a polarizing microscope (BX51; Olympus Corporation, Tokyo, Japan). The root diameter (RD) (mm), vascular bundle diameter (VBD) (mm), cortex thickness (CT) (mm), xylem area (XA; cm^2^), number of vessels (NV) were measured from one root cross section order sample. In addition, the stele: root diameter ratio (VBD/RD) is calculated to represent the ratio of root diameter used for resource transportation. And we calculated cortex ratio (C/R; cortex area / root cross section area) from the measured (Natsuko et al., 2019).

### Soil sampling, physical, and chemical properties analysis

First of all, the button thermometer (DS1921G) was covered with a sealed bag and buried in the soil to continuously measure the soil temperature, set to record every 2 h. Then, we used the ring-knife method (inner diameter 5 cm) to collect soil samples 50 cm from the trunk of each standard tree for the determination of soil bulk density (SBD) and soil porosity (SP). Finally, after removing the litter and humus layer on the soil surface, we collected three topsoil samples with a 5 cm diameter drill at a depth of 0–20 cm at the fine root sampling point for the determination of soil chemical properties. This depth was chosen since fine roots are mainly confined to the upper soil, and the relationship between fine roots and physical and chemical properties of soil should be stronger than that of deep soil (He et al., 2022). After collection, all soil samples were stored in a refrigerator at low temperature (0–4°C) and transported to the laboratory as soon as possible for further analysis.

In the laboratory, we mixed all soil samples were mixed into one composite sample per tree to determine the physical and chemical properties of soil (He et al., 2022). First, the physical properties of the soil: SBD and SP were determined by the ring-knife method (Yokobe et al., 2021); SW was determined by determining water loss (Yokobe et al., 2021). Then, remove impurities (such as rocks, plant litter) and determine the chemical properties of the soil: soil total nitrogen (STN) was determined by the semi-micro Kjeldahl digestion method (Sinha et al., 1986); soil total phosphorus (STP) was determined by the sodium-hydroxide fusion molybdenum antimony anti-coloring method (Fisher et al., 1998; Hogan et al., 2021); soil total potassium (STK) was determined by the sodium-hydroxide melting-flame atomic absorption spectrophotometry method (Hogan et al., 2021); soil alkali-hydrolyzed nitrogen (SAN) was determined by the alkali-solution diffusion method (Hogan et al., 2021); soil available phosphorus (SAP) was determined by the sodium bicarbonate extraction-molybdenum-antimony colorimetric method (Cao et al., 2021); soil available potassium (SAK) was determined by the neutral ammonium acetate extraction-atomic absorption spectrophotometry determination method (Hogan et al., 2021); soil organic carbon (SOC) was determined by the potassium dichromate acid oxidation-external heating method (Curtin and Smillie, 1986).

### Data analysis

First, the Shapiro–Wilk test and Levene’s test were used to determine the normality and homogeneity of variance for each group of variables, respectively. And use Excel software (Version 2019, Microsoft Office, United States) to manage, sort and calculate the data (He et al., 2022).

Second, the SPSS software (Version 25.0, SPSS Inc., Chicago, IL, United States) was used to analyze the anatomical characteristics of fine roots of different root orders (RD, VBD, VBD/RD, CT, C/R, XA, NV) and soil physical and chemical properties (SOC, STN, STP, STK, SAN, SAP, SAK, SW, SBD and SP). The differences in fine roots anatomical structure, soil physical and chemical properties, and root orders among the four test sites were analyzed by one-way ANOVA, followed by Duncan’s multiple comparison method (*p* < 0.05). Two-way ANOVA was used to analyze the effects of soil factors, root order and their interactions on fine root anatomical structure and nutrients.

Finally, the redundancy analyses (RDA) (Li et al., 2020) was performed to analyze the relationships in fine roots anatomical structure, soil environmental factors, and nutrients content by using Canoco software (version 5.0).

## Results

### Characteristics of fine root anatomical structure in the different city sites

First, we analyze whether different soil conditions have an impact on the anatomical structure of each level of fine roots. Our results show that both the four experimental sites and root order have a significant influence on the anatomical structure of fine roots (*p* < 0.01, Table 2). However, the interaction between them has different influences on them, specifically, it has extremely significant influence on VBD and VBD/RD (*p* < 0.01, Table 3), significant influence on XA and NV (*p* < 0.05, Table 2), but no influence on RD, CT and C/R (*p* > 0.05, Table 2). That is to say, the interaction between sites and root order mainly affects the stele, but not the cortex. So the interaction between them mainly affects the transport capacity of fine roots, but has little effect on the absorption function.

**Table 2:** Variance analysis of the influence of site and roots order on the anatomical structure of fine roots.

Source of variation	Diameter (mm)	Vascular bundle diameter (mm)	Stele: root diameter ratio	Cortex thickness (mm)	Cortex proportion	Xylem area (cm^2^)	Number of vessels
Site	12.189^**^	24.583^**^	124.547^**^	11.787^**^	10.24^**^	28.123^**^	64.704^**^
Root order	231.582^**^	832.255^**^	599.023^**^	7.964^**^	15.462^**^	474.491^**^	1922.003^**^
Site × root order	1.36NS	2.324^*^	1.931^*^	0.613NS	0.624NS	14.811^**^	22.992^**^

NS indicated *p* > 0.05, ^*^and ^**^indicate significance at *p* < 0.05 and *p* < 0.01, respectively.

**Table 3:** *Cupressus funebris* anatomical structure of fine root in the first to fifth order roots among four test sites.

Index	Root order	Site			
		SN	MY	DY	GA
RD (mm)	1	550.52 ± 78.63cA	450.36 ± 48.1cB	432.99 ± 49.56 dB	526.25 ± 25.69dA
	2	615.36 ± 19.39cA	505.21 ± 32.48cB	522.6 ± 51.65cdB	587.96 ± 19.39dA
	3	700.96 ± 97.9cA	470.91 ± 82.16cB	614.4 ± 107.13cB	686.08 ± 5.25cA
	4	997.92 ± 273.41bA	830.4 ± 119.5bA	860.49 ± 133.49bA	936.16 ± 119.03bA
	5	1718.68 ± 36.89aA	1332.8 ± 293.47aB	1456.14 ± 170.43aAB	1676.69 ± 34.32aA
VBD (mm)	1	138.27 ± 10.38eB	169.98 ± 18.36dA	157.32 ± 9.27eA	138.77 ± 7.51dB
	2	206.5 ± 33.52dA	227.44 ± 21.22cA	218.79 ± 18.03dA	164.45 ± 15.39 dB
	3	288.9 ± 25.72cB	282.66 ± 38.05cB	389.96 ± 90.37cA	238.98 ± 30.11cB
	4	593.68 ± 63.27bC	661.15 ± 23.9bB	752.84 ± 12.5bA	554.67 ± 28.77bC
	5	1189.03 ± 78.76aAB	1220.66 ± 87.29aA	1189.58 ± 25.96aAB	1075.06 ± 76.62aB
VBD/RD	1	0.25 ± 0.03cB	0.38 ± 0.02eA	0.37 ± 0.02cA	0.26 ± 0.01cB
	2	0.27 ± 0.14cB	0.45 ± 0.02dA	0.42 ± 0.02cA	0.28 ± 0.019cB
	3	0.43 ± 0.02bB	0.57 ± 0.04cA	0.61 ± 0.06bA	0.36 ± 0.06bB
	4	0.66 ± 0.07aB	0.74 ± 0.01bA	0.81 ± 0.04aA	0.64 ± 0.02aB
	5	0.69 ± 0.056aBC	0.79 ± 0.01aAB	0.82 ± 0.09aA	0.64 ± 0.04aC
CT (mm)	1	179.62 ± 8.97aA	155.53 ± 24.84aBC	137.5 ± 14.99aC	165.19 ± 24.49aAB
	2	178.3 ± 12.28aA	161.67 ± 16.63aA	157.22 ± 24.21aA	176.63 ± 18.02aA
	3	194.32 ± 21.68aA	167.98 ± 3.27aB	158.1 ± 17.91aB	183.07 ± 5.94aAB
C/R	1	179.62 ± 8.97aA	0.86 ± 0.01cB	0.86 ± 0.012aB	0.93 ± 0.01aA
	2	178.3 ± 12.28aA	0.81 ± 0.012bB	0.81 ± 0.02aB	0.92 ± 0.01aA
	3	194.32 ± 21.68aA	0.65 ± 0.06aA	0.59 ± 0.27aA	0.85 ± 0.02bA
XA (cm^2^)	1	181.78 ± 47.68cB	229.30 ± 36.27cAB	266.84 ± 26.37cA	196.11 ± 40.08cB
	2	850.09 ± 259.92cA	917.21 ± 407.88cA	1140.56 ± 78.61cA	824.26 ± 217.63cA
	3	11947.28 ± 5960.15cA	14153.3 ± 9334.86cA	16225.46 ± 8274.36cA	6747.45 ± 829.36cA
	4	73590.73 ± 10728.3bB	86180.26 ± 11051.30bB	113716.7 ± 23349.24bA	69736.75 ± 5304.81bB
	5	205039.15 ± 64862.08aB	345172.24 ± 47495.18aA	383233.07 ± 60423.98aA	185150.09 ± 68712.41aB
NV	1	9 ± 1.20dB	16 ± 5.35cA	13 ± 3.63dAB	10.43 ± 2.23cB
	2	22.5 ± 6.346dB	45 ± 13.589cA	36.25 ± 3.454dA	17 ± 3.493cB
	3	228.86 ± 19.95cB	273.67 ± 9.03cA	302.67 ± 36.96cA	154.33 ± 16.73cC
	4	643.25 ± 97.93bBC	765.67 ± 68.09bAB	871.75 ± 125.96bA	503 ± 30.38bC
	5	2081.2 ± 78.10aB	2,856 ± 535.29aA	3,051 ± 220.52aA	1970.67 ± 232.64aB

Different lowercase letters in the same column indicated that there were significant differences among different root orders (*p* < 0.05). Different capital letters of the same trade indicate that there are significant differences among different locations (*p* < 0.05). RD means fine root diameter, VBD means vascular bundle diameter, VBD/RD means stele: root diameter ratio, CT means cortex thickness, C/R means cortex ratio, XA means xylem area, NV means number of vessels.

In the four experimental sites, RD, VBD, VBD/RD, CT, C/R, XY and VE gradually increased with the increase of root order. For the first-order fine roots of *C. funebris*, there are significant differences in RD, VBD, VBD/RD and C/R between MY, DY and SN, GA (*p* < 0.05, Table 3). For the second-order fine roots, RD, VBD/RD, C/R and NV are significantly different between MY, DY and SN, GA (*p* < 0.05, Table 3). For the third-order fine roots, RD, VBD/RD and C/R are significantly different between MY, DY and SN, GA (*p* < 0.05, Table 3). For the fourth-order fine roots, VBD and VBD/RD were significantly different between MY, DY and SN and GA, and the contents of XA and NV in SN were significantly higher than those in the other three regions (*p* < 0.05, Table 3). For the fifth-order fine roots, RD is significantly different between MY and DY, SN, GA, VBD is significantly different between MY and GA, XA and NV are significantly different between MY, DY and SN, GA (*p* < 0.05, Table 3). The differences in anatomical structure of fine root sequences between the four sites indicated that fine roots had heterogeneous plasticity.

### Correlation between fine root anatomical structure and soil environmental factors

Secondly, the physical and chemical properties of soil in the four experimental sites are different after our analysis. PCA analysis showed that the soil nutrients were the highest in DY, followed by MY and SN, and the lowest in GA (He et al., 2022). The details is shown in Supplementary Table S1.

RDA showed that the amount of soil environmental factors (STN, STP, STK, SAN, SAP, SAK, SOC, SW, SBD and SP) explaining fine root anatomical structure (RD, VBD, VBD/RD, CT, C/R, XA, NV) can show the adaptive strategies of plants in a particular environment.

**Figure 2:**
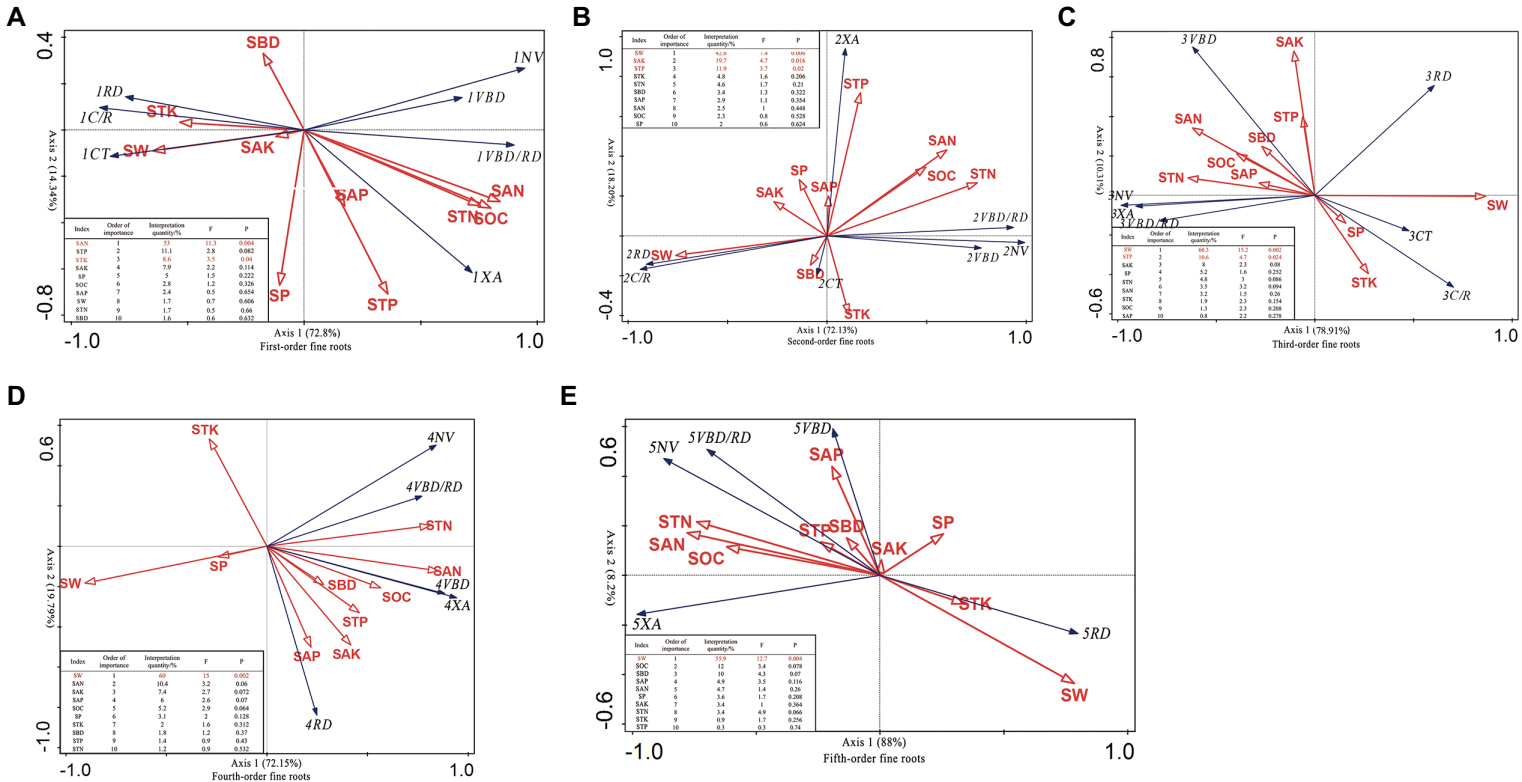
RDA analysis between soil environmental factors and fine root anatomical structure of *C. funebris*. First-order fine roots **(A)**, second-order fine roots **(B)**, third-order fine roots **(C)**, fourth-order fine roots **(D)**, fifth-order fine roots **(E)**. The blue arrows represent the anatomical features of fine roots, including (RD, VBD, VBD/RD, CT, C/R, XA, NV); Red arrows represent the cedar wood and soil environmental factors, including (SOC, STN, STP, SAN, SAK, SAP and SAK, SW, SBD, SP). RD, means fine root diameter, VBD, means vascular bundle diameter, VBD/RD, means stele: root diameter ratio, CT, means cortex thickness, C/R, means cortex ratio, XA, means xylem area, NV, means number of vessels; SOC, means organic carbon, STN, means Total nitrogen, STP, stands for total phosphorus, STK, stands for total potassium, SAN, stands for alkali hydrolyzed nitrogen, SAP, stands for available phosphorus, SAK, stands for available potassium; SW, stands for soil moisture, SBD, stands for soil bulk density, and SP, stands for soil porosity. The letters in this figure indicated the same meaning as the chart below.

For the first-order fine roots, vascular bundle and xylem are positively correlated with N and SOC, negatively correlated with K and SW, while the cortex is just the opposite. And the diameter was positively correlated with K, SW and SBD, and negatively correlated with N, P and organic carbon (Figure 2A). For second-order fine roots, vascular bundles and xylem were positively correlated with N and SOC, and negatively correlated with K, SBD and SW, while the cortical tissue was on the contrary. And the diameter is negatively correlated with SW, SBD and SP (Figure 2B). For third-order fine roots, vascular bundles, the tissue and xylem were positively correlated with N, P, SOC and SBD, and negatively correlated with SW. The cortical tissue is just the opposite. The root diameter was positively correlated with SW and negatively correlated with N and SOC (Figure 2C). The fifth-order fine roots are very similar to the fourth-order fine roots. There was no cortical tissue in all of them. Vascular bundle tissue and xylem were positively correlated with N, P, K, SOC and SBD, and negatively correlated with SW. The difference is that the diameter of fourth-order fine roots are also positively correlated with N, P, and the diameter of fifth-order fine roots are positively correlated with K and SW (Figures 2D,E). These results show that the anatomical structure of fine roots at all orders is closely related to soil factors, showing specific adaptation strategies of fine roots.

Finally, the importance of soil environmental factors to the anatomical structure of 1–5 order fine roots was ranked. The anatomical structure of first-order fine roots of *C. funebris* was mainly affected by SAN, STK (*p* < 0.05), accounting for 53 and 8.6% of the total information of soil environmental factors, respectively. Second-order fine roots were most affected by SW, SAK and STP (*p* < 0.05), accounting for 42.6, 19.7 and 11.9%, respectively. Third-order fine roots were mainly affected by SW and STP (*p* < 0.05), accounting for 60.3 and 10.6%, respectively. SW had the greatest effect on fourth- to fifth-order fine roots (*p* < 0.05), accounting for 60 and 55.9%. From this, we can conclude that the fine roots of high-order roots are mainly affected by soil moisture, while the fine roots of low-order roots are greatly affected by soil nutrients (N, P, K).

## Discussion

### Anatomical structure of fine roots under different soil conditions

In response to the change of soil environment, the anatomical structure of fine root shows plasticity in the trade-off between the acquisition and protection of soil resources (soil water and nutrients; Liu et al., 2019; Yang et al., 2019), which supports our first hypothesis. Specifically, the anatomical characteristics of roots vary according to root sequence (Wang et al., 2019), soil nutrients (Chen et al., 2010), soil bulk density (Eissenstat et al., 2015; Freschet et al., 2017) and soil biological community (Hishi, 2007).

Our results show that the anatomical structure of fine roots of *C. funebris* shows obvious geographical differences with the change of soil environment. Cortical thickness is one of the important indexes to measure the ability of root absorption and radial transport (Guo et al., 2008). In this study, the areas with high STN and SAN (such as DY and MY, Supplementary Table S1) exerted thicker cortex, but smaller vascular bundle diameter and xylem area (Table 3). This is due to the decrease of cortical cell diameter or the number of cortical cell layers under high N condition (Oscar et al., 2001). It is also worth noting that, contrary to the response of cortical tissue, the diameter or cross-sectional area of fine roots increased under the condition of high N (Stefan et al., 2001). The fine root vascular bundle diameter, xylem tissue and vessel number reflect the root transport capacity. The higher the value, the stronger the fine root transport capacity and the stronger the ability to resist adverse environmental factors (Guo et al., 2008; Jia et al., 2013). Therefore, we found that in the environment with relatively high soil nitrogen content, the transport capacity of fine root was stronger than that in the environment with relatively low nitrogen content. At the same time, there is a correlation between the anatomical structure and physiological variation of fine roots (Weemstra et al., 2016). Several studies have shown that fine roots can respond to stressful environments by altering their anatomical structure, including the development of larger vascular bundles in saturated soils (Yamauchi et al., 2021) and the formation of aerenchyma (Kotula et al., 2015). This means that the development of cortex tissue of *C. funebris* may be relatively better in the environment of soil nutrient deficiency, while the development of vascular tissue of *C. funebris* may be relatively better in the environment of soil nutrient abundance.

Diameter is the most important morphological feature of root system with a secondary predictor of root anatomical structure variation (Gu et al., 2014). According to the growth law of plants, the thickness of diameter is determined by both the thickness of cortex and the diameter of vascular bundles. Most studies have shown that there is a significant positive correlation between fine root anatomical structure (cortex thickness, vascular bundle diameter) and root diameter in tropical, subtropical and temperate forests (Kong and Ma, 2014; Liu et al., 2018). In this study, fine roots exerted larger diameter and thicker cortex in SN and GA, especially in 1- to 2-order fine roots (Table 3). We speculate that the reason may be related to the higher soil moisture content in SN and GA (Supplementary Table S1). Due to the root anatomical and physiological activities are closely related (McCormack et al., 2015), fine root morphological changes could be the result of water demand and physical changes (Kong et al., 2017). Studies have shown that root diameter decreases organization to reduce the main reason is that the root cortex (Kong et al., 2017, 2019). Therefore, in areas with higher soil moisture content (such as SN and GA), the metabolic activity of fine roots is more vigorous, and to ensure the rapid acquisition and transportation of water and nutrients, the thickness of the cortex is also larger, and the fine roots are thicker. In addition, a thicker diameter may be built to maintain its absorptive capacity for a longer time (Zadworny et al., 2016), and the long-term investment in organizational construction and maintenance is relatively low (Valverdea-Barrantes et al., 2020).

At the same time, soil moisture was the main soil environmental factor affecting the anatomical structure of second- to fifth-order fine roots (Figure 2). Vascular tissue was negatively correlated with soil moisture, while cortical tissue was positively correlated with soil moisture (Figure 2). The function of the xylem conduit is to collect and transport water and minerals from the root to the above ground (Ouyang et al., 2020). On the one hand, the water in the soil enters the roots through xylem “pipes” (Warren et al., 2015). And through the availability of soil water, root growth and microbial activity can be regulated, which indirectly affects the C cycle of underground soil (Curiel et al., 2003; Weltzin et al., 2009). On the other hand, drought-tolerant plants promote the formation of periderm in fine roots in higher root order (Blokhina et al., 2003). *C. funebris* has strong drought tolerance. In the environment with relatively little soil moisture (DY and MY), fine roots can promote the thickening of periderm thickness to reduce water loss and promote the development of xylem structure, which can transport water needed by various organs more effectively. Thus, the growth and development of root system is affected by environmental conditions have plasticity, thus to better face the changes in the environment.

### Response of fine root order function in different soil environments

Root order rather than diameter has been shown to be a better representative of root function (Weemstra et al., 2016). The differences in root anatomy among different root sequences enable them to perform different ecological functions, such as nutrient acquisition and transport (Yu et al., 2022). Usually, the five-level root order can be divided into two groups: the root whose main function is to absorb resources and the root that serves functions other than absorption (Guo et al., 2008). Absorptive fine roots are mainly involved in acquiring and absorbing soil resources, while transportable fine roots mainly play the functions of structure and transportation, and have some additional storage capacity (McCormack et al., 2015). The development of root function from absorption to transport may be at the cost of reducing the thickness of cortical tissue, indicating the difference of absorption function from high to low and transport function from low to high along the root order. Changes in fine root function usually represent different strategies or adaptations of plants to various environmental conditions (de la Riva et al., 2021).

Although the root order is different, plants have been shown to respond to the soil environment. With regard to the classification of fine roots, generally speaking, the first- to third-order fine roots of plants are low-order roots (absorbing roots) and fourth- to fifth-order fine roots are high-grade roots (transport roots; Wang et al., 2019). It has been realized that this classification method is too general to take into account the impact of soil environmental changes on the anatomical structure of fine roots, and cannot explain the changes in fine root functional groups (Freschet and Roumet, 2017). Recent studies have further emphasized that the structural and morphological characteristics of different root sequences can change independently of each other, making woody plants adapt to different environments (McCormack et al., 2020).

With the change of soil environment, the function of different root orders is not fixed. For example, in the site with the highest STN (that is DY), the fine root diameter and the vascular bundle diameter of 3-order fine roots was high (Table 3). On the one hand, N was previously thought to play a similar core role in the characteristic tissues of roots, supporting metabolic activities (including nutrient and water transport, enzyme function and mycorrhizal; Ma et al., 2018); On the other hand, soil moisture is vital for the diffusion of N to the root surface (Warren et al., 2015). Therefore, we speculate that due to the low soil moisture content in DY (Supplementary Table S1), fine roots may not be able to transport enough N to the aboveground parts of plants through diffusion. Since, by increasing the diameter of fine roots and vascular bundles,3-order fine roots can also obtain enough N. And there are studies that show that as soil N increases, fine roots transport function increases by decreasing the thickness of cortex (Chen et al., 2010). Therefore, in order to fix nitrogen and absorb more water in arid soil, we analyzed that the 3-order fine roots have a tendency to evolve into transport roots. However, we observed that in areas with low N content (such as SN), the thickness of the 3-order fine roots cortex were significantly higher than that of the other three experimental areas (Table 3). In other words, in the soil with low N content, the 3-order fine roots of the plant may perform the function of absorption. The transformation of the function of 3-order fine roots under different soil conditions may be driven by environmental pressure to evolve fine roots that can adapt to different soils, which is a more competitive strategy for fine roots to compete for soil nutrients. Another key factor behind the occurrence of functional slippage in 3-order fine roots may be that the stele and cortical tissue are allometric (Valverdea-Barrantes, 2022). It was found that the ratio of the cortex (occupied root cross-sectional area) to the stele (occupied root cross-sectional area) increased as the root diameter increased will achieve a better balance between nutrient absorption and transport (Zhou et al., 2021). The allometric relationship followed by the stellars and cortex helps us to better understand the strategies for the acquisition and conservation of third-order fine root resources and their adaptation to different environments.

In addition, we analyzed that in our four different sites, 1- and 2-order fine roots showed intact cortex and low stele proportion, so we defined them as absorbent roots. Similarly, 4- and 5-order fine roots showed no cortex and obvious secondary development, so we defined them as transport roots (Guo et al., 2008). Of course, we also wonder whether the soil environment changes greatly, will the function of the 2-order and 4-order fine roots also change? For example, from tropical rain forest to desert or north forest. The root economic spectrum (RES) advocates a trade-off between maximizing access to resources and improving productivity, or maximizing protection of resources and prolonging life (Carmona et al., 2021; de la Riva et al., 2021). Studies have shown that the root life with high maximum absorption rate is shorter (Weemstra et al., 2016). From the perspective of cost-effectiveness, it is difficult to explain the mismatch between the fact that all 1- to 4-order fine roots are absorbing roots or 2- to 5-order fine roots are transporting roots and the conservation of root phylogeny. Therefore, 2-order fine roots will not become transport roots, and 4-order fine roots will not become absorbent roots. If the function of the 2-order and 4-order fine roots is also changed, then the absorption and transport functions of the plant will lose coordination. However, differences in the anatomical structure of fine roots at different levels affect the efficiency of absorption of nutrients and water in fine roots (1- and 2-order) and transportation of nutrients and water in fine roots (4- and 5-order).

These results further support the view that the responses of fine root anatomical structures with different root orders to soil environmental factors may not be consistent. This structure of the root system is helpful for the lower roots to more easily acquire and effectively utilize the resources in the soil environment, and the higher roots to more easily and efficiently transport water and nutrients to all parts of the plant, so as to maintain the demand for water and nutrients and normal growth of the plant.

## Conclusion

We used several typical indexes (diameter, cortical tissue, vascular tissue and xylem) to evaluate the variation of fine root anatomical structure and the division of fine root functional groups with different root order under different soil conditions. Our conclusion is that soil environmental factors may influence the anatomical structure of fine roots with different root orders. First of all, for higher order roots, soil moisture (SW) was the most important factor; for lower order roots, soil nutrients (N, P, K) were more important. Then, our study fine roots are plastic in their response to local environmental conditions during development: low soil moisture and nutrient content promoted the development of fine root cortex, while high soil moisture and nutrient content promoted the development of vascular bundles. Finally, there was obvious heterogeneity in anatomical structure and function of fine roots with different root sequences. As fine roots adapt to different soil environments, the division of fine root functional groups may not fixed. Although different soil environments did not change the functional group characteristics of 1–2 order fine roots as absorption roots and 4–5 order fine roots as transport roots, their absorption and transport efficiency would be affected. The exception is the 3-order root, which we define as a transitional root because it may be biased in favor of absorbing or transporting roots under different circumstances. In short, fine roots respond to changes in soil environment by changing different anatomical structures, and their corresponding functions will also change, which is the adaptive strategy of fine roots.

The next step would continue to study the effects of soil environment on fine roots. For example, the nutrients and water physiology of fine roots are worth studying.

## Data availability statement

The original contributions presented in the study are included in the article/Supplementary material, further inquiries can be directed to the corresponding author.

## Author contributions

TL, JR, and CF planned and designed the research. TL, JR, WH, YW, XWe, XWa, and MY performed experiments, conducted fieldwork, and analyzed data. TL wrote the manuscript. GC, KZ, GH, XL, and CF contributed to the revision. All authors contributed to the article and approved the submitted version.

## Conflict of interest

The authors declare that the research was conducted in the absence of any commercial or financial relationships that could be construed as a potential conflict of interest.

## Publisher’s note

All claims expressed in this article are solely those of the authors and do not necessarily represent those of their affiliated organizations, or those of the publisher, the editors and the reviewers. Any product that may be evaluated in this article, or claim that may be made by its manufacturer, is not guaranteed or endorsed by the publisher.

## Supplementary material

SUPPLEMENTARY FIGURE S1 One of treated fine roots of 1–5-ordee fine roots.

SUPPLEMENTARY FIGURE S2 Anatomical structure of 1–5-order fine roots of *Cupressus funebris* from the 4 test sites. Magnification of anatomical structure: the images of 1- to 3-ordee fine roots were taken at ×10, and those of 4- and 5-order fine roots were taken at ×4.
