# Supplementary figures and images for: Anatomical structure interpretation of the effect of soil environment on fine root function
Source: Front Plant Sci. 2022 Aug 30;13:993127. doi: 10.3389/fpls.2022.993127 (PMC9470114; doi:10.3389/fpls.2022.993127)

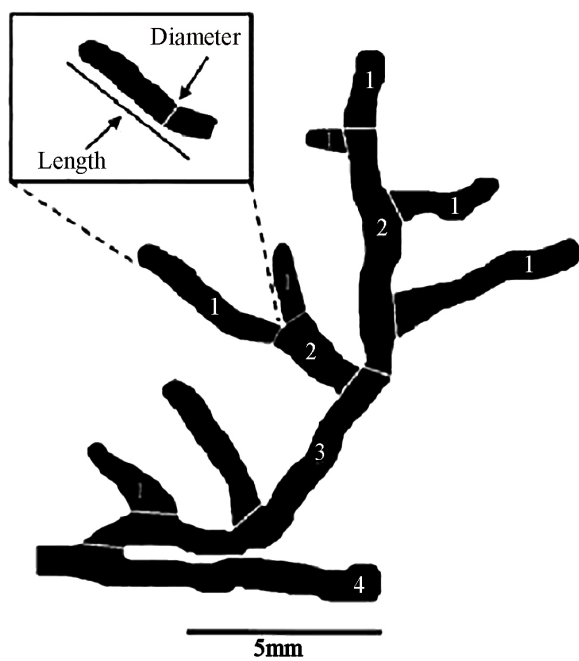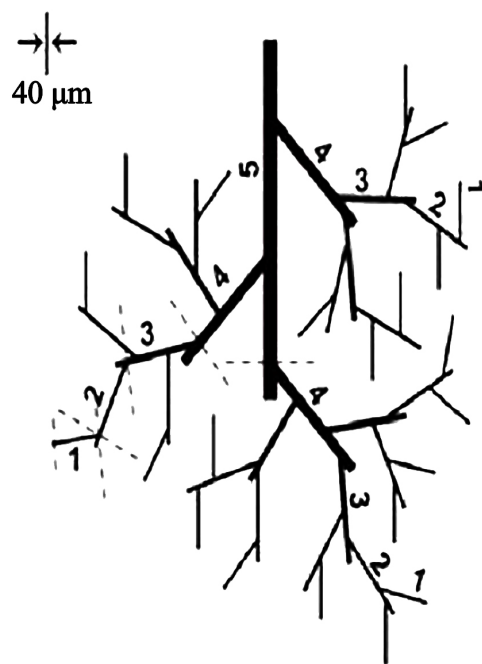

Supplement: SUPPLEMENTARY FIGURE S1 — One of treated fine roots of 1–5-ordee fine roots. [file Image_1.pdf]

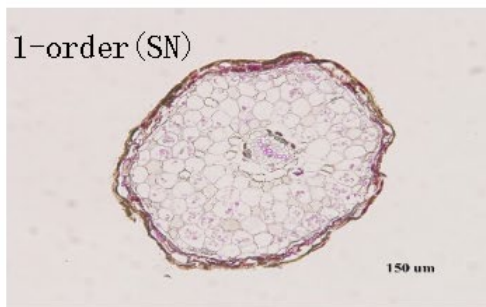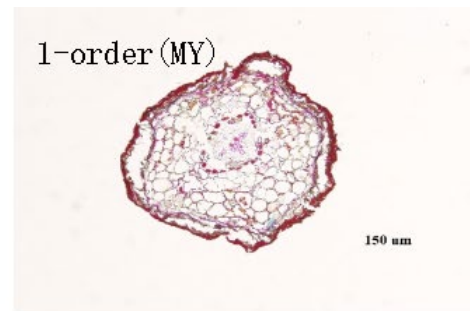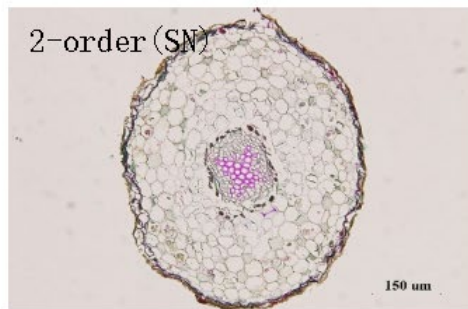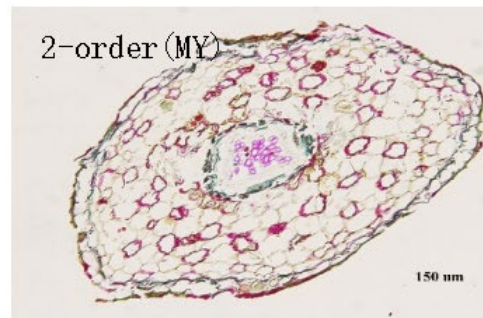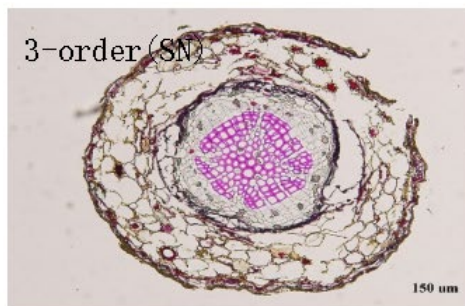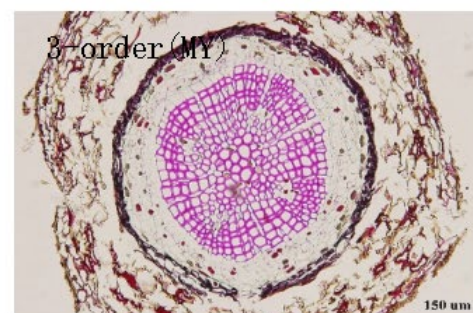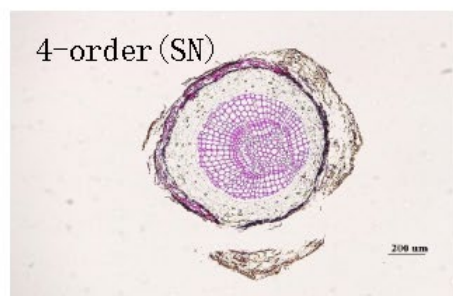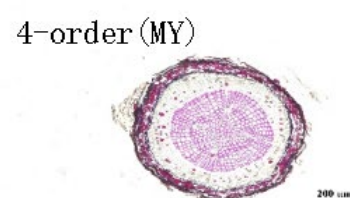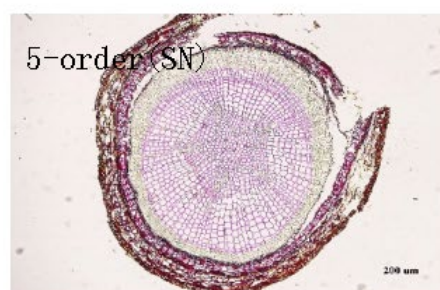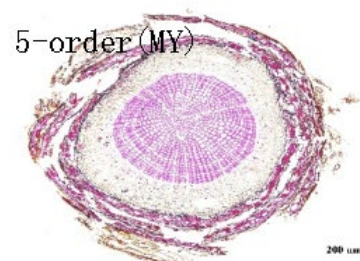

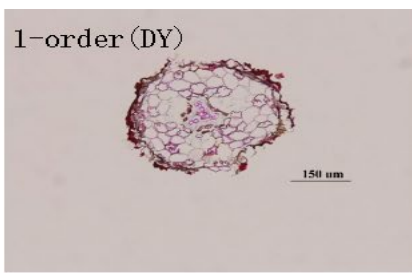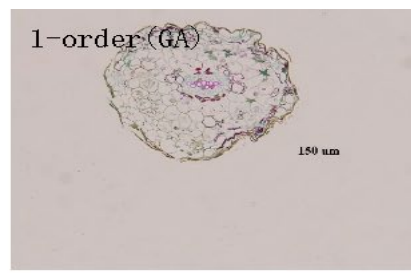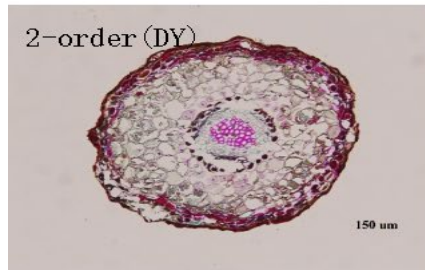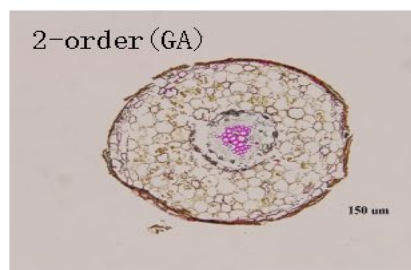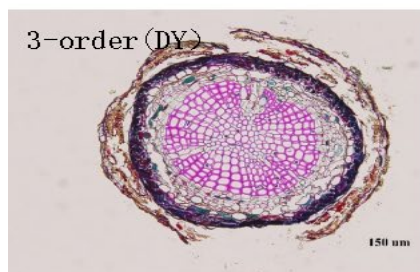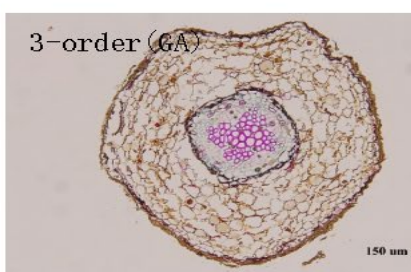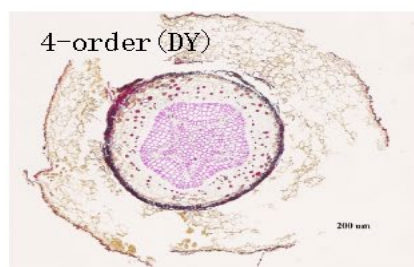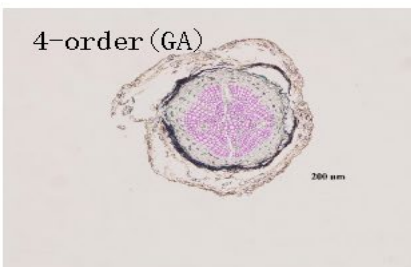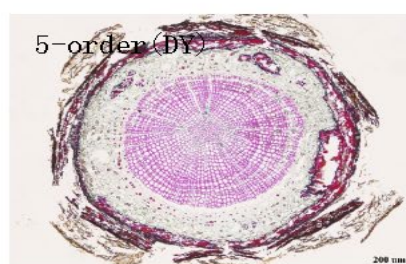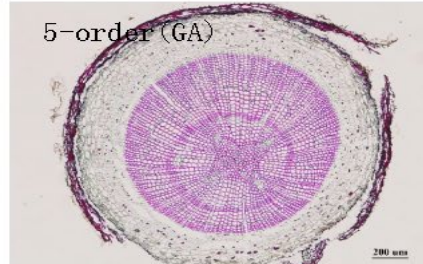

Supplement: SUPPLEMENTARY FIGURE S2 — Anatomical structure of 1–5-order fine roots of Cupressus funebris from the 4 test sites. Magnification of anatomical structure: the images of 1- to 3-ordee fine roots were taken at ×10, and those of 4- and 5-order fine roots were taken at ×4. [file Image_2.pdf]
