# Supplementary material for: Anatomical structure interpretation of the effect of soil environment on fine root function
Source: Front Plant Sci. 2022 Aug 30;13:993127. doi: 10.3389/fpls.2022.993127 (PMC9470114; doi:10.3389/fpls.2022.993127)
Supplement: Supplementary file 3 [file Table_1.docx]

**Table S1 Physical and chemical properties of forest soil in four test sites**

| Index | Site | | | |
| --- | --- | --- | --- | --- |
|  | Suining | Mianyang | Deyang | Guangan |
| Organic carbon（g·kg-1） | 27.97±2.86c | 47.35±1.52b | 61.97±2.90a | 47.07±1.89b |
| Total nitrogen (mg·kg-1) | 0.94±0.10c | 1.64±0.37b | 2.34±0.23a | 1.09±0.14bc |
| Total phosphorus (mg·kg-1) | 0.39±0.01a | 0.39±0.01a | 0.41±0.013a | 0.40±0.018a |
| Total potassium (mg·kg-1) | 32.05±1.14a | 31.55±12.8a | 21.66±2.60a | 26.63±3.31a |
| Alkaline nitrogen (mg·kg-1) | 52.23±5.12d | 82.52±1.42b | 124.99±4.97a | 66.55±4.31c |
| Available phosphorus (mg·kg-1) | 6.16±0.41a | 6.01±3.97a | 6.84±0.30a | 5.96±0.15a |
| Available potassium (mg·kg-1) | 104.6±25.44ab | 47.75±8.47c | 108.59±13.53a | 66.91±5.20bc |
| Soil moisture (%) | 21.67±0.59b | 19.18±0.23c | 17.43±0.53d | 27.98±0.26a |
| Soil temperature(℃) | 21.7±0.04b | 22.3±0.05a | 20.51±0.05c | 17.95±0.03d |
| Soil bulk density (g·cm-3) | 1.55±0.15a | 1.27±0.07ab | 1.40±0.08ab | 1.12±0.16b |
| Soil porosity (%) | 43.12±5.25a | 43.59±5.88a | 46.60±3.46a | 50.52±3.98a |
