# Supplementary material for: Anatomical structure interpretation of the effect of soil environment on fine root function
Source: Front Plant Sci. 2022 Aug 30;13:993127. doi: 10.3389/fpls.2022.993127 (PMC9470114; doi:10.3389/fpls.2022.993127)
Supplement: Supplementary file 4 [file Table_2.docx]

**Table S2 List of abbreviations**

| Full Name | Abbreviations | Full Name | Abbreviations |
| --- | --- | --- | --- |
| Suining | SN | Soil Total Nitrogen | STN |
| Deyang | DY | Soil Alkali hydrolyzed nitrogen | SAN |
| Mianyang | MY | Soil Total phosphorus | STP |
| Guangan | GA | Soil Available phosphorus | SAP |
| Fine Root Diameter | RD | Soil Total potassium | STK |
| Vascular Bundle Diameter | VBD | Soil Readily available potassium | SAK |
| Vascular Bundle/ Root Diameter | VBD/RD | Soil Organic carbon | SOC |
| Cortex Thickness | CT | Soil moisture | SW |
| Cortex ratio | C/R | Soil temperature | ST |
| Xylem Area | XA | Soil bulk density | SBD |
| Number of Vessels | NV | Soil porosity | SP |
